# Knock-Out of ACBD3 Leads to Dispersed Golgi Structure, but Unaffected Mitochondrial Functions in HEK293 and HeLa Cells

**DOI:** 10.3390/ijms22147270

**Published:** 2021-07-06

**Authors:** Tereza Daňhelovská, Lucie Zdražilová, Hana Štufková, Marie Vanišová, Nikol Volfová, Jana Křížová, Ondřej Kuda, Jana Sládková, Markéta Tesařová

**Affiliations:** 1 Department of Paediatrics and Inherited Metabolic Disorders, Charles University, First Faculty of Medicine and General University Hospital in Prague, 128 01 Prague, Czech Republic; tereza.danhelovska@vfn.cz (T.D.); lucie.zdrazilova@lf1.cuni.cz (L.Z.); Hana.Stufkova@vfn.cz (H.Š.); Marie.Rodinova@vfn.cz (M.V.); Nikol.Volfova@lf1.cuni.cz (N.V.); Jana.Krizova@vfn.cz (J.K.); jsladkova@centrum.cz (J.S.); 2 Institute of Physiology, Academy of Sciences of the Czech Republic, 142 00 Prague, Czech Republic; Ondrej.Kuda@fgu.cas.cz

**Keywords:** ACBD3, mitochondria, cholesterol, Golgi, OXPHOS, knock-out

## Abstract

The Acyl-CoA-binding domain-containing protein (ACBD3) plays multiple roles across the cell. Although generally associated with the Golgi apparatus, it operates also in mitochondria. In steroidogenic cells, ACBD3 is an important part of a multiprotein complex transporting cholesterol into mitochondria. Balance in mitochondrial cholesterol is essential for proper mitochondrial protein biosynthesis, among others. We generated ACBD3 knock-out (ACBD3-KO) HEK293 and HeLa cells and characterized the impact of protein absence on mitochondria, Golgi, and lipid profile. In ACBD3-KO cells, cholesterol level and mitochondrial structure and functions are not altered, demonstrating that an alternative pathway of cholesterol transport into mitochondria exists. However, ACBD3-KO cells exhibit enlarged Golgi area with absence of stacks and ribbon-like formation, confirming the importance of ACBD3 in Golgi stacking. The glycosylation of the LAMP2 glycoprotein was not affected by the altered Golgi structure. Moreover, decreased sphingomyelins together with normal ceramides and sphingomyelin synthase activity reveal the importance of ACBD3 in ceramide transport from ER to Golgi.

## 1. Introduction

Humans express seven highly conserved Acyl-CoA-binding proteins (ACBD1–ACBD7). A common feature of this protein family is the ACB domain, responsible for the binding of long-chain fatty Acyl-CoA esters. ACBD3 is the largest protein of this family, consisting, apart from the ACB domain, of a coiled-coil domain in the middle and a Golgi dynamics (GOLD) domain on the C terminus. The GOLD domain is responsible for multiple protein interactions and may be used to stabilize peripheral membrane proteins at intracellular membranes. As reported in The Human Protein Atlas [1], ACBD3 is highly expressed in some organs of the digestive system, brain, prostate, placenta, and bone marrow; medium expression is characteristic of male and female reproductive tissues (for a complete summary, see Table A1). According to its antibody validation profile, ACBD3 is localized in Golgi (The Human Protein Atlas) and is a membrane-bound or membrane-associated protein. Inferring from sequence similarity, it is probably also localized in mitochondria [1,2,3]. The MitoCarta predictions for ACBD proteins are summarized in Table A2. 

According to published research [4,5,6,7,8,9], ACBD3 is a protein localized in endoplasmic reticulum (ER), Golgi, mitochondria, plasma membrane, and cytosol. ACBD3 participates in multiple protein–protein interactions and has various functions: a Golgi–ER tether or a Golgi scaffold protein, in vesicle trafficking (sphingolipid transport), mitochondrial cholesterol transport/steroid synthesis, or in the regulation of cellular iron uptake. Moreover, it serves as a host interaction protein for the replication of multiple members of the picornavirus family (multiple ACBD3–protein interactions and its functions are well summarized in [10] In this work, we focused on the role of ACBD3 in mitochondria.

The function of ACBD3 in mitochondria has generally been studied in relation to the transport of cholesterol into mitochondria for steroidogenesis [11]. However, mitochondrial cholesterol is not only necessary for synthesis of steroids, oxysterols, and hepatic bile acids; it is also an integral part of mitochondrial membranes. Mitochondrial DNA (mtDNA) exists in nucleoprotein structures called nucleoids, which are associated with mitochondrial membranes and facilitate mtDNA maintenance and gene expression [12]. Nucleoids differ in protein components, depending on their functions—replication, translation, and repair [13]. Membrane-associated replication platforms, containing the major replication proteins, are abundant in cholesterol, and a disruption of the cholesterol homeostasis, for example by gene silencing of *ATAD3* (ATPase family AAA domain-containing protein 3), impairs mtDNA topology and mitochondrial protein synthesis [13,14,15,16].

The mechanism of transporting cholesterol into mitochondria is still not well known. Free cholesterol is nearly insoluble in water and therefore depends on transport via cholesterol-binding proteins [17]. In steroidogenic cells, StAR (steroidogenic acute regulatory protein) is involved in the transport of cholesterol from lipid droplets and from the ER to the outer mitochondrial membrane (OMM). StAR is a part of a multiprotein complex, but the exact composition of this complex and the mechanism of cholesterol transport are still debated [11]. The group led by V. Papadopoulos described a multiprotein complex (transduceosome) formed of StAR, VDAC1, TSPO, ACBD3, PKARIα (type I PKA), ATAD3, and CYP11A1 (mito cyt P450) [6,18]. The ACBD3 protein was described as an interacting partner between TSPO and PKARIα [5], serving as an A-kinase-anchoring protein for PKARIα, which has a role in phosphorylation and activation of StAR. Recently, a new role of ACBD3 in the mitochondrial retrograde response, induced by mitochondrial dysfunction, has been described [19]. ACBD3, together with TSPO and PKA, is indispensable in adaptation to stress, via retro-communication with the nucleus [19].

As mentioned above, ACBD3 has an apparently important function in transporting cholesterol into mitochondria. A disruption of cholesterol transport into mitochondria could affect proper mitochondrial replication and protein biosynthesis, and thus lead to secondary mitochondrial defects. The aim of this study was to characterize the impact of a complete absence of the ABCD3 protein on mitochondrial cholesterol level and related changes in mitochondrial energy metabolism: level of mtDNA, representation of mtDNA encoded proteins, mitochondrial function (mitochondrial respiration), production of reactive oxygen species (ROS), and mitochondrial ultrastructure in HEK293 and HeLa cells. In addition, we focused on Golgi structure, the representation of Golgi proteins, and the glycosylation pattern of the LAMP2 glycoprotein. Last but not least, we determined the level of various lipids in ACBD3 knock-out (ACBD3-KO) HEK293 and HeLa cell lines and their isolated mitochondria. Our results emphasize the role of ACBD3 protein in Golgi stacking and suggest the alternative pathway of transporting cholesterol into mitochondria, independent of the ACBD3 protein.

## 2. Results

### 2.1. Localisation of ACBD3 Protein

**Figure 1:**
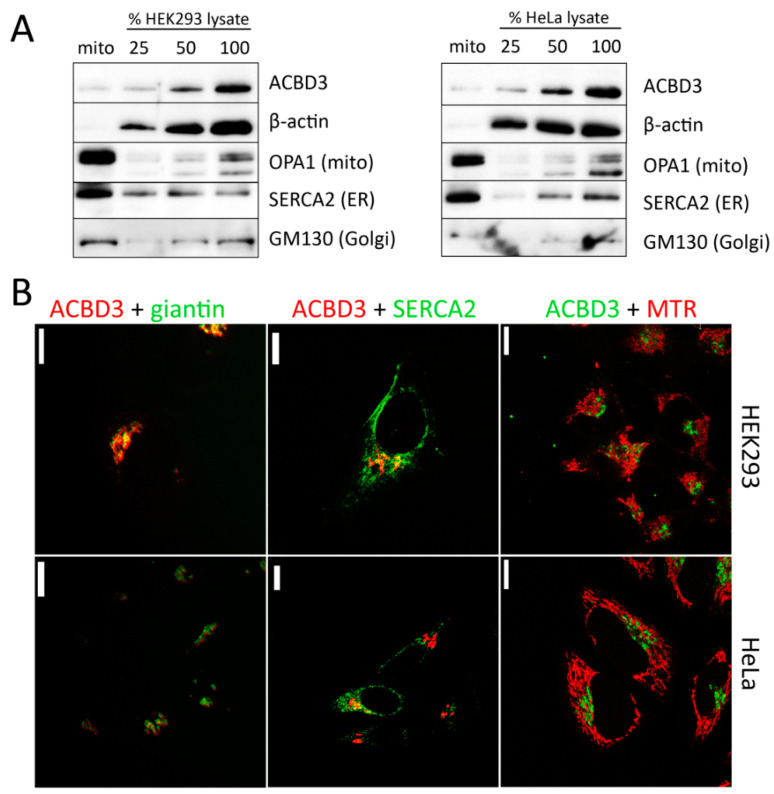
Localization of ACBD3 protein in HEK293 and HeLa WT cells. (**A**) Characterization of mitochondrial fraction and whole cell lysate by SDS-PAGE/WB. The mitochondrial fraction reveals only a slight signal of ACBD3, but the mitochondrial fraction is enriched by endoplasmic reticulum (ER, SERCA2 antibody) and also a slight signal of Golgi (GM130). (**B**) Confocal microscopy of HEK293 WT and HeLa WT cells. The cells were immunolabeled by ACBD3 antibody and, where indicated, by specific markers for Golgi (giantin), ER (SERCA2), and mitochondria (MitoTracker Red (MTR)). Overlay of signals was found only between ACBD3 and giantin (Golgi). Scale bar 10 µm.

To reveal the localization of ACBD3 in HEK293 wild-type (WT) and HeLa WT cells, we used the Mitochondria Isolation Kit based on the MACS technology, which yields high-purity mitochondria [20]. In the mitochondrial fraction, we observed approximately 15% of the ACBD3 signal compared to the cell lysate and insignificant contamination of cytosol (β-actin), but the mitochondrial fraction showed an enrichment by ER, in addition to a faint signal of the Golgi protein GM-130 (Figure 1A). Therefore, we are not able to conclude if ACBD3 is localized in the mitochondria from this result. To determine the localization of the ACBD3 protein, we performed confocal microscopy of HEK293 and HeLa WT cells, focusing on selected organelles (Golgi, ER, and mitochondria) as well. Signal overlay was observed only between ACBD3 and giantin (Figure 1B). These findings suggest that ACBD3 localizes primarily to Golgi in HEK293 and HeLa cells and its interactions with mitochondria might be transient.

### 2.2. Characterization of ACBD3-KO HEK293 and HeLa Cells

**Figure A1:**
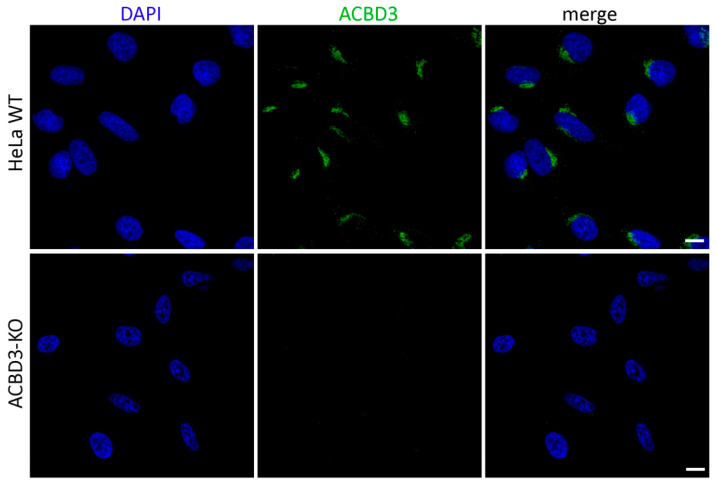
Confocal immunofluorescence images of ACBD3 antibody in HeLa WT and ACBD3-KO cell lines. Scale bar 10 μm.

We created three HEK293 ACBD3-KO cell lines and one HeLa ACBD3-KO cell line. Together, all four ACBD3-KO clones (HEK293 ACBD3-KO 24, 59, and 87; HeLa ACBD3-KO B3) showed consistent results across a broad range of analyses. Immunofluorescence images of ACBD3-KO B3 are displayed in Figure A1.

Firstly, we focused on mitochondrial and Golgi ultrastructure, followed by an assessment of the Golgi area, steady-state level of representative Golgi proteins, and the glycosylation of the LAMP2 glycoprotein in HEK293 and HeLa ACBD3-KO. Then, we carried out several well-established analyses to study mitochondrial OXPHOS in ACBD3-KO cells: the representation of OXPHOS protein complexes and selected mitochondrial proteins, mitochondrial respirometry, ROS production, and the relative mtDNA amount. Finally, we performed a lipidomics analysis of ACBD3-KO cells.

#### 2.2.1. Mitochondria and Golgi Ultrastructure

**Figure 2:**
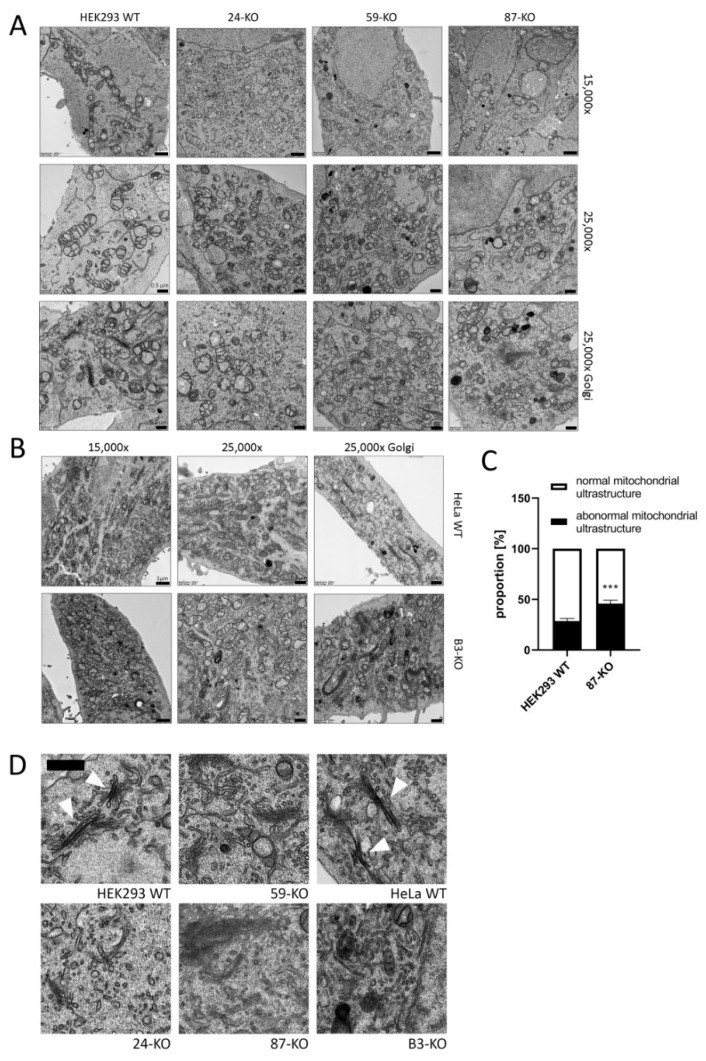
Transmission electron microscopy (TEM) of the ultrastructure of mitochondria (15,000× (scale bar 1 µm) and 25,000× (scale bar 0.5 µm)) and the Golgi apparatus (25,000×) in (**A**) HEK293 ACBD3-KO cells and (**B**) HeLa ACBD3-KO cells. (**C**) Quantification of the mitochondria with normal and abnormal ultrastructure in HEK293 WT and 87-KO. More than 300 mitochondria from 27 pictures per cell line were used to determine statistical significance by Mann–Whitney t-test. Error bar represents SEM, *p* < 0.001 (***). (**D**) Detail of Golgi structure. Arrows point to normal Golgi structure in HEK293 and HeLa WT. In ACBD3-KO cells, normal Golgi structure with stacked cisternae was barely found. However, we detected multiplied vesicles in the Golgi area. Scale bar 0.5 µm.

Significant changes in mitochondrial ultrastructure were found in multiple cellular disturbances [21,22]. Therefore, we focused on the study of mitochondrial ultrastructure by Transmission Electron Microscopy (TEM). Overall, mitochondrial ultrastructure was not altered in ACBD3-KO cells (Figure 2A,B). In most of the cells, mitochondria exhibited intact structure with normal cristae formation comparable with control samples. However, a significantly increased proportion of mitochondria with abnormal structure was observed in 87-KO (Figure 2A and quantification in Figure 2C). Since the ACBD3 protein primarily acts in Golgi, we also examined the structure of this organelle. We did not see the regular ribbon and stacked cisternae formation, but we observed a significantly increased amount of vesicles in the Golgi area (Figure 2A,B and detail in Figure 2D). This confirms the important role of ACBD3 in maintaining the structure of Golgi.

#### 2.2.2. Golgi Assessment (Golgi Area Measurement, Golgi Proteins, and Glycosylation of the LAMP2 Glycoprotein) in ACBD3-KO Cells

**Figure 3:**
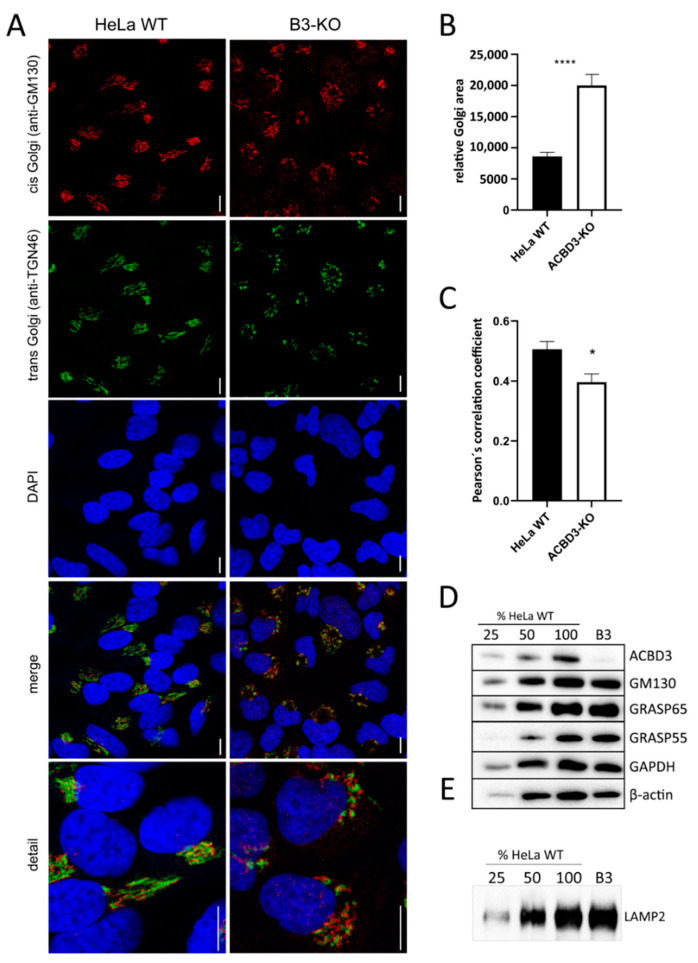
Golgi assessment in ACBD3-KO cells. (**A**) Confocal immunofluorescence images of cis- (GM130) and trans-Golgi markers (TGN-46) in HeLa WT and ACBD3-KO. In ACBD3-KO, Golgi structure is extremely fragmented, whereas control cells exhibit a compact structure, typical for Golgi. Scale bar 10 µm. (**B**) Measurement of relative Golgi area in HeLa WT and ACBD3-KO from more than 60 cells was performed using ImageJ. Error bars represent SEM. A Mann–Whitney test was used to determine statistical significance, *p* < 0.0001 (****). (**C**) Pearson’s correlation coefficient was applied to quantify GM130 and TGN46 colocalization using LAS X software (Leica, Wetzlar, Germany). Error bars represent SEM. A Mann–Whitney test was performed to determine statistical significance, *p* < 0.05 (*). (**D**) Steady-state level of selected Golgi proteins in control and ACBD3-KO cells determined by SDS-PAGE/WB. The numbers 25, 50, and 100 demonstrate loading dose of protein. Relative signal intensity was normalized to the intensity of loading control (GAPDH) by densitometric analysis. None of the analyzed Golgi proteins showed any significance change in protein amount but the level of β-actin was altered in ACBD3-KO cells. (**E**) The mobility assay of the LAMP2 glycoprotein did not reveal any alteration in ACBD3-KO cells. The numbers 25, 50, and 100 demonstrate loading dose of protein.

**Figure A2:**
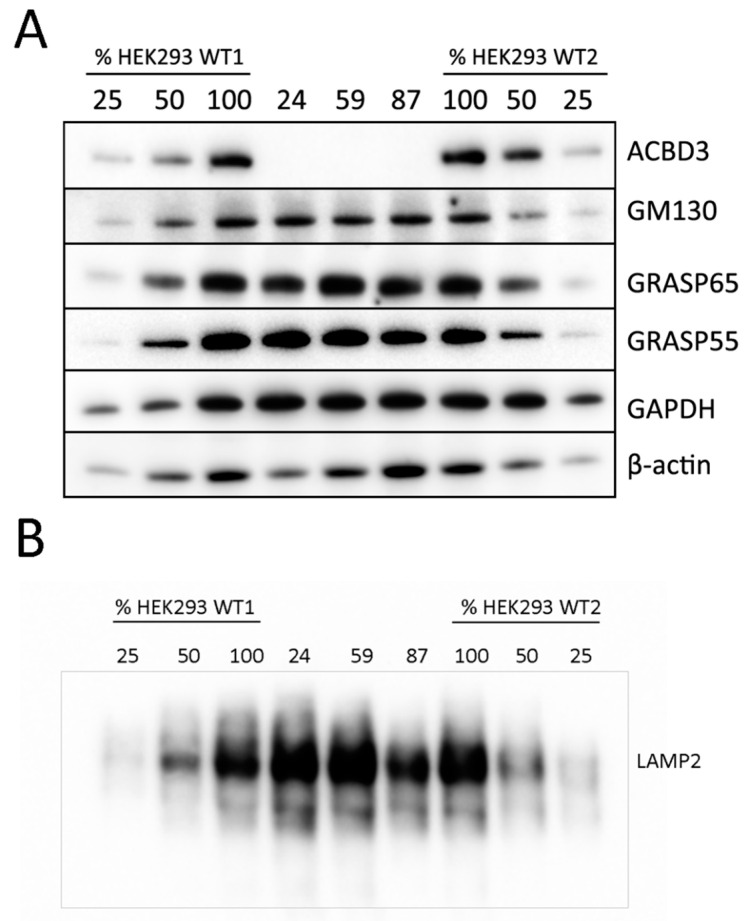
(**A**) Western blots of selected Golgi proteins in HEK293 ACBD3-KO cells. (**B**) Glycosylation of LAMP2 glycoprotein in HEK293 ACBD3-KO cells.

Due to altered Golgi structure on TEM (Figure 2D), we performed immunofluorescence labeling of cis- (GM130) and trans-Golgi (TGN46) markers. The ACBD3-KO cells showed an extremely fragmented and disorganized Golgi structure (Figure 3A). The relative Golgi area was notably expanded (Figure 3B) and the cis- and trans-Golgi signals did not co-localize (Figure 3C). Then, we determined the level of selected Golgi proteins involved in the maintenance of Golgi structure. The amount of all analyzed Golgi protein remained in the control range, but the level of β-actin changed (Figure 3D and Figure A2A). Because the Golgi structure was significantly disturbed, we examined the glycosylation pattern of the LAMP2 glycoprotein using the mobility shift assay, but found the pattern comparable with control samples (Figure 3E and Figure A2B).

#### 2.2.3. OXPHOS Complexes and Subunits, Mitochondrial Respiration, ROS Production, and Relative mtDNA Amount

**Figure 4:**
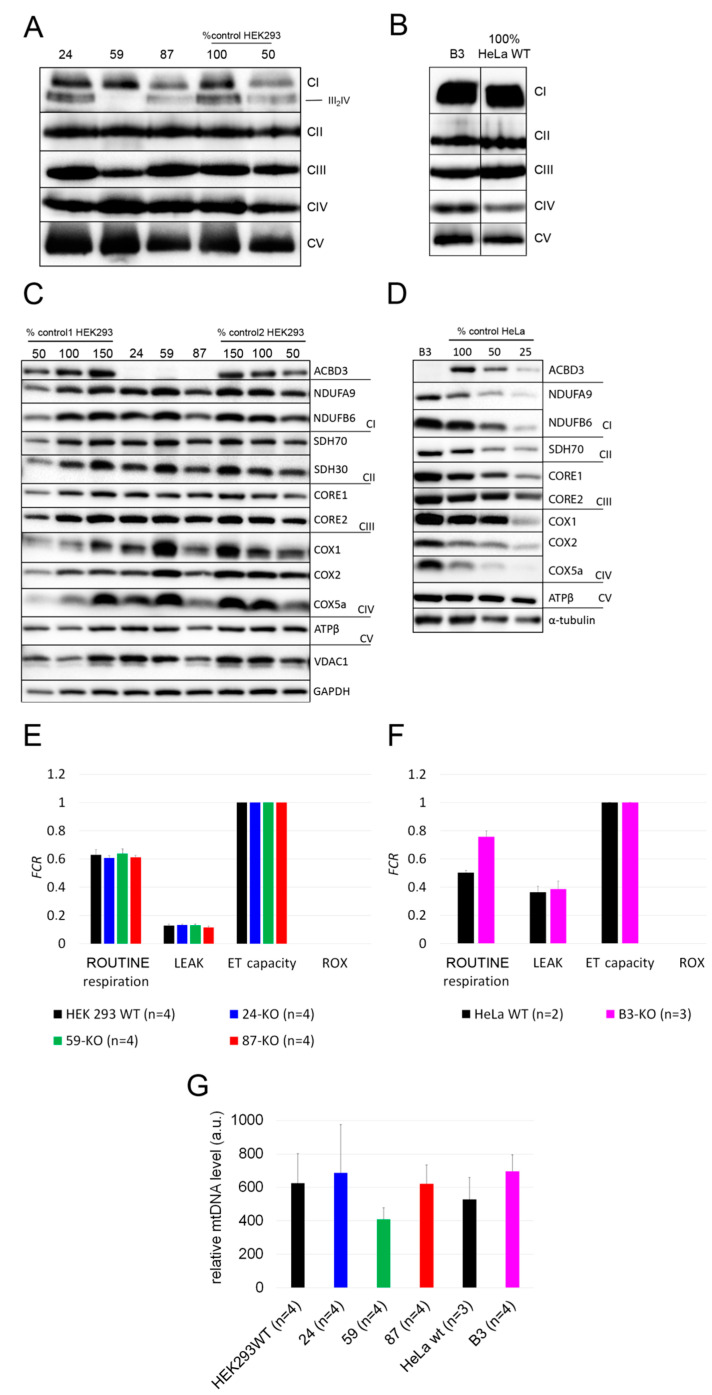
Mitochondrial investigations in ACBD3-KO cells. (**A**) Steady-state level of OXPHOS protein complexes in isolated mitochondria from HEK293 and (**B**) HeLa analyzed by BN-PAGE/WB. The numbers 50 and 100 show the loading dose of protein. Relative signal intensity was normalized to the intensity of complex II by densitometric analysis. (**C**) Steady-state level of selected OXPHOS protein subunits in HEK293 and (**D**) HeLa analyzed by SDS-PAGE/WB. The numbers 25, 50, 100, and 150% indicate loading dose of protein. Relative signal intensity of individual antibodies was normalized to the intensity of loading control (GAPDH and α-tubulin, respectively) by densitometric analysis. (**E**) High-resolution respirometry in HEK293 and (**F**) HeLa cell lines. ROUTINE respiration shows physiological respiration, LEAK shows proton leak after the inhibition of ATP synthase by oligomycin. Residual oxygen consumption (ROX) and electron transfer capacity (ET capacity) represent the minimal and maximal nonphysiological values of respiration, which are set on 0.0 and 1.0 in *FCR*. (**G**) A comparison of mtDNA level across ACBD3-KO cell lines and controls. ‘n’ represents the number of independently analyzed samples per each group. Error bars represent SD.

**Figure A3:**
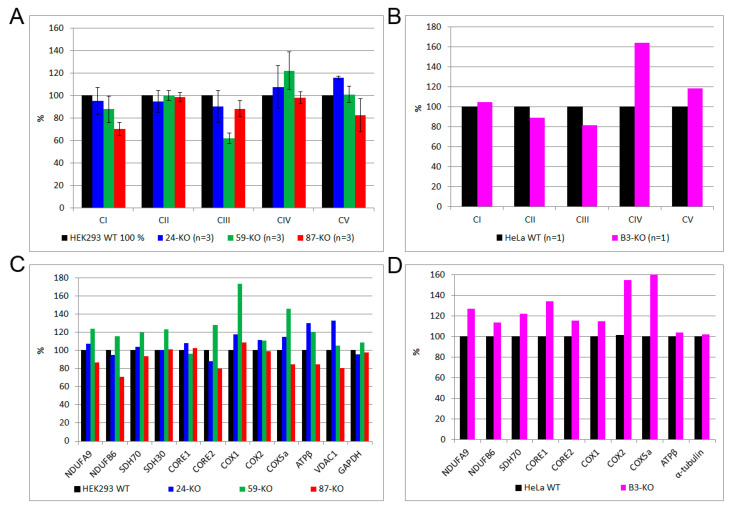
Quantification of Western blots from Figure 4. (**A**) BN-PAGE/WB of HEK293 ACBD3-KO, (**B**) BN-PAGE/WB of HeLa ACBD3-KO, (**C**) SDS-PAGE/WB of HEK293 ACBD3-KO, and (**D**) SDS-PAGE/WB of HeLa ACBD3-KO. ‘n’ represents number of independent analyses per sample.

**Figure A4:**
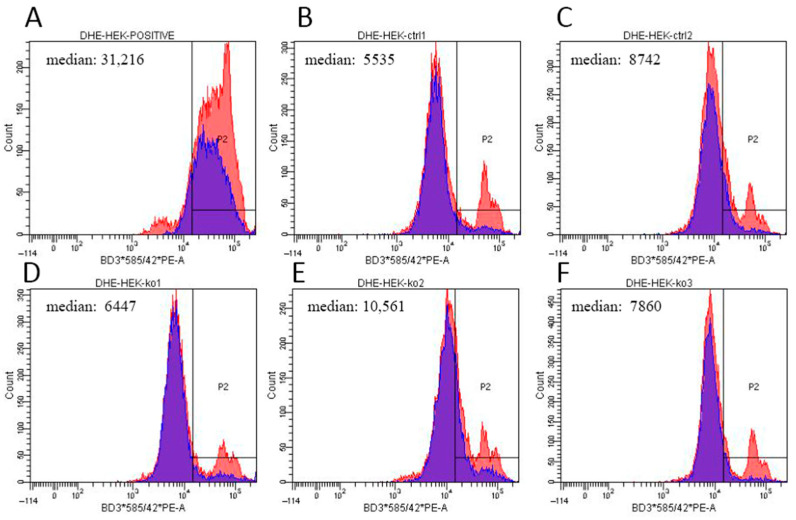
ROS detection by flow cytometry after DHE staining. Histogram plots show the fluorescent signal distribution in (**A**) a positive control treated with 100 μM menadione, two control cell lines ((**B**) HEK293 WT1 and (**C**) WT2), and ACBD3-KO cell lines (**D**) 24-KO, (**E**) 59-KO, and (**F**) 87-KO. *x*-axis: fluorescence intensity; *y*-axis: cell count. Blue populations represents cells without ROS-positive signal in control samples; medians are figured in each plot. Slight increase in ROS production was observed in ACBD3-KO cell line 59 (median 10,561). ROS production in the other two ACBD3-KO cell lines remained in control range.

**Figure A5:**
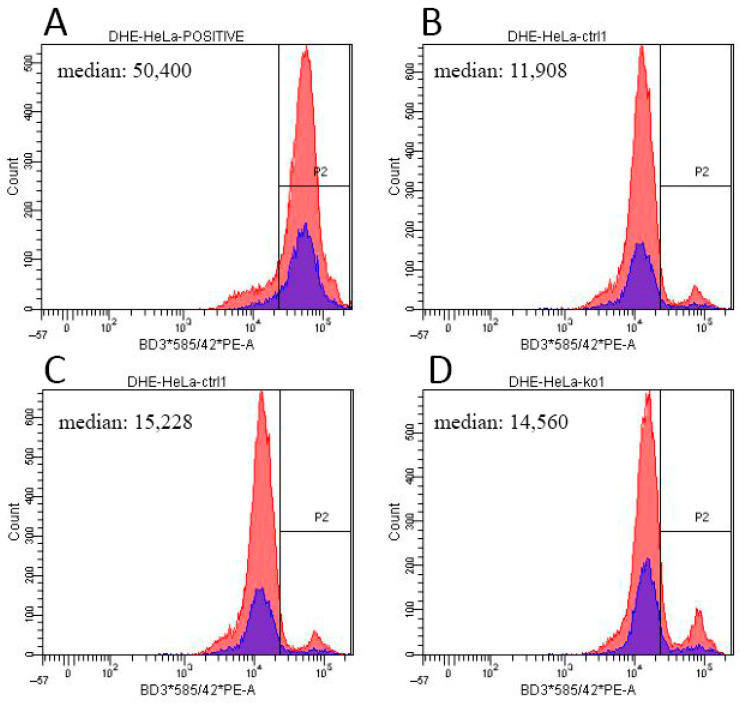
ROS detection by flow cytometry after DHE staining. Histogram plots show the fluorescent signal distribution in (**A**) a positive control treated with 100 μM menadione, two control cell lines ((**B**) HeLa WT1 and (**C**) WT2), and ACBD3-KO cell line (**D**) B3-KO. *x*-axis: fluorescence intensity; *y*-axis: cell count. In case of HeLa ABCD3-KO, ROS production remained similar to controls.

We determined the steady-state levels of OXPHOS complexes in isolated mitochondria from ACBD3-KO cell lines. Overall, the amount of OXPHOS protein complexes was not significantly disturbed. We only observed a partial decrease in the complex III level in 59-KO (62% of control value), a mildly decreased amount of complex I in 87-KO (70% of control value), and an increased level of complex IV in B3-KO (184% of control value; Figure 4A,B; quantification in Figure A3A,B). Following the analysis of OXPHOS protein complexes, we determined the amount of selected OXPHOS protein subunits (Figure 4C,D; quantification in Figure A3C,D). We found marked changes in the case of complex IV subunits (encoded by both mtDNA and nuclear DNA) in cell lines 59-KO (COX1 160% of control value and COX5a 134% of control value) and B3-KO (COX2 at 152% of control value and COX5a at 250% of control value), but they had no impact on the assembly of complex IV (Figure 4A,B) and the formation of OXPHOS supercomplexes (data not shown). Altogether, only a mild alteration in the levels of several subunits were found, while in general, the amounts of OXPHOS protein subunits did not significantly change across ACBD3-KO cell lines (Figure 4C,D). Apart from OXPHOS protein subunits, we focused also on proteins of the transduceosome. Selected proteins (VDAC1, TSPO, ACBD1, and ANT) were not altered in ACBD3-KO cells (data not shown). Mitochondrial respiration in most ACBD3-KO cells corresponded with controls in all states, with the exception of B3-KO cells, where the ROUTINE respiration, controlled by cellular energy demand and turnover, was increased in comparison to the control, indicating possible cellular stress (Figure 4E,F). Next, we analyzed ROS production in all ACBD3-KO cell lines to determine oxidative stress. ROS production remained within the control range in all ACBD3-KO cells except 59-KO, where a small amount of ROS-positive cells was detected (Figure A4 and Figure A5). The analysis of mtDNA content did not reveal any alteration across the ACBD3-KO cell lines (Figure 4G). Based on these results, neither mitochondrial OXPHOS, nor the mtDNA amount, seem significantly altered in ACBD3-KO cells.

#### 2.2.4. Lipidomics in ACBD3-KO Cells

**Figure 5:**
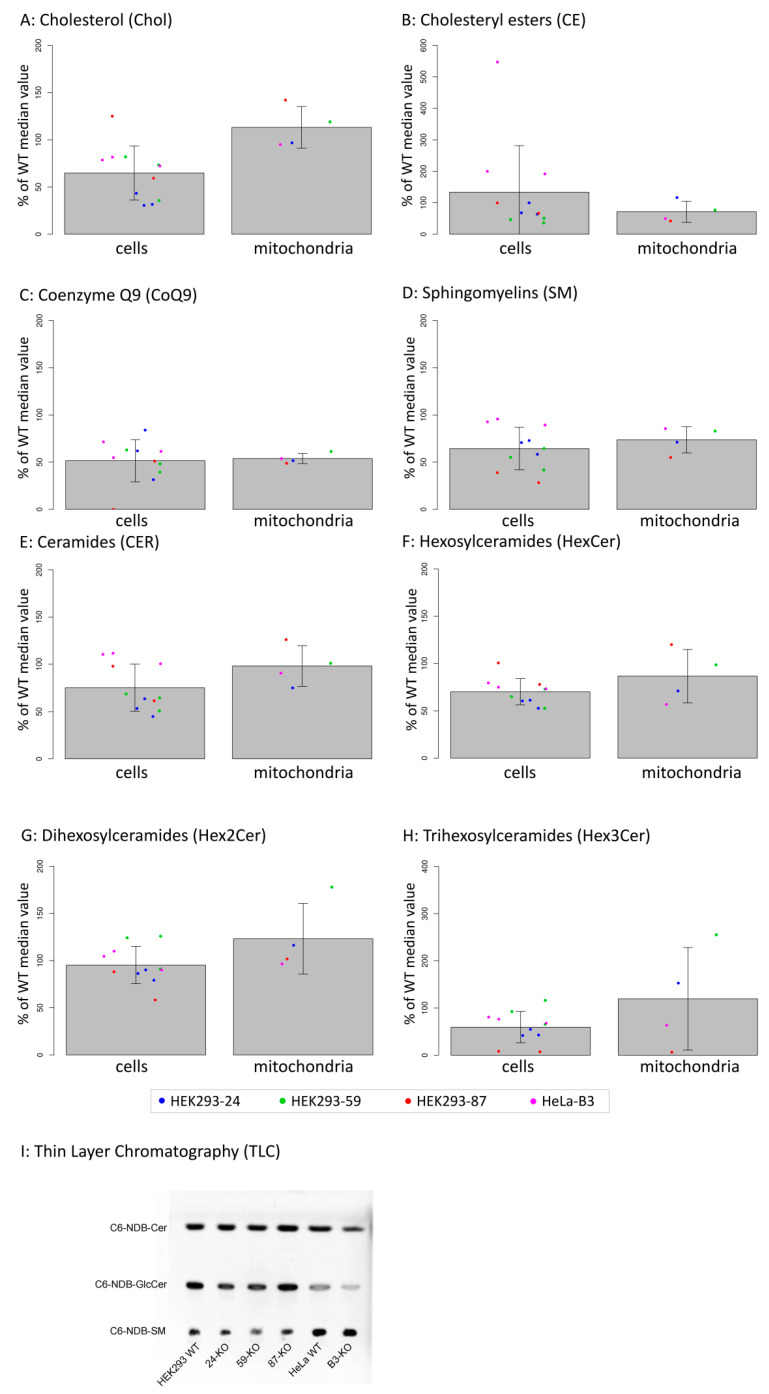
(**A**–**H**): Lipidomics analysis in whole cells and in isolated mitochondria of ACBD3-KO cells displayed as percentage of control values. Cells were measured in triplicate (duplicate in the case of 87-KO), dots represent individual obtained values, bar charts represent mean values, and errors bars represent SD. Significance of differences between WT and KO in cells and in mitochondria are summarized in Table 1. Linear mixed-effects models were used for statistical analysis. (**I**) In situ analysis of sphingomyelin synthase (SMS) and glucosylceramide synthase (GCS) activities using fluorescent-labeled ceramide (C6-NBD-Cer). Activities of SMS and GCS are visualized as the amount of synthesized products, sphingomyelin (C6-NBD-SM), and glucosylceramide (C6-NBD-GlcCer), respectively. Quantification of TLC results is shown in Figure A6.

**Figure A6:**
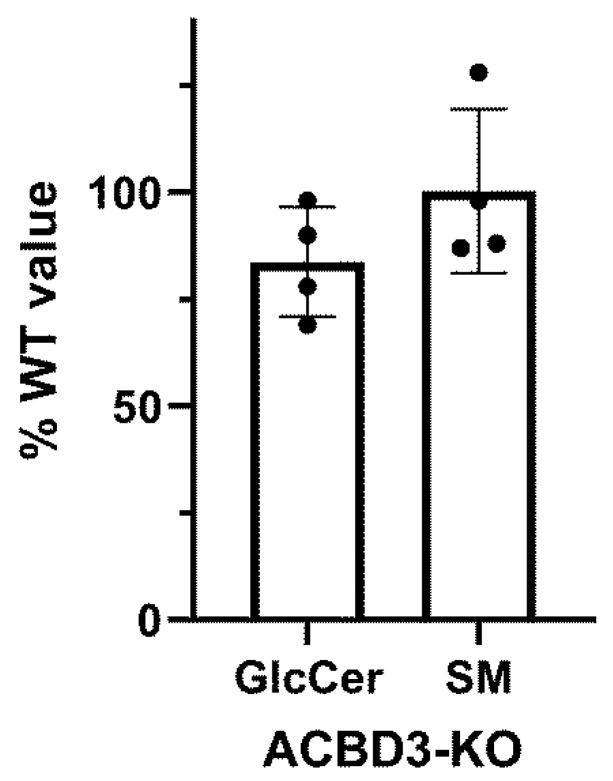
Quantification TLC results from Figure 5I. Relative signal intensity of glucosylceramide synthase (GlcCer) activity and sphingomyelin synthase (SM) activity were normalized to the intensity of ceramide by a densitometric analysis. Each dot represents value for particular ACBD3-KO. Values are displayed as a percentage of control value.

To evaluate our hypothesis that the ACBD3 protein transports cholesterol into mitochondria and the prediction that its absence will disrupt cholesterol levels in mitochondria, we performed a lipidomics analysis in ACBD3-KO cells as well as in isolated mitochondria (Figure 5A–H, statistics in Table 1). The level of cholesterol did not significantly differ in cells nor in the isolated mitochondria, but the cholesteryl esters were altered significantly. Interestingly, the level of coenzyme Q9 (CoQ9) was significantly lower in both whole cells and mitochondria compared to control samples. Moreover, we also observed significantly decreased levels of sphingomyelins (SM), but normal ceramides and hexosyl ceramides levels in both the cells and the mitochondria compared to controls. Due to the decreased sphingomyelins, we performed an in situ measurement of sphingomyelin synthase (SMS) and glucosylceramide synthase (GCS) activity by quantifying the conversion of C6-NBD-ceramide, a fluorescent ceramide analogue, to C6-NBD-SM (SMS activity) and C6-NBD-GlcCer (GCS activity), respectively, followed by TLC detection [23]. The assay did not reveal any significant changes in SMS and GCS activity across the ACBD3-KO cell lines (Figure 5I, quantification in Figure A6).

## 3. Discussion

### 3.1. Altered Golgi Structure in ACBD3-Deficient Cells

In general, ACBD3 is primarily associated with Golgi and to this date, its interactions with several distinct proteins in different parts of Golgi have been described [4,7,8,9,24,25,26]. Similarly to others, using immunofluorescence and WB, we showed localization of ACBD3, particularly in Golgi. Functions of these ACBD3-containing protein complexes range from maintenance of the Golgi structure, membrane trafficking, and glycosphingolipid metabolism to regulation of *de novo* fatty acid synthesis or apoptosis [10]. Yue et al. [7] described ACBD3 as a part of multiprotein cisternal adhesion complex (ACBD3-TBC1D22-GRASP55-golgin45), where the exact role of ACBD3 in Golgi stacking remains unclear. Our results from TEM and immunofluorescence imaging show that the absence of ACBD3 significantly affects the structure of the Golgi complex. In ACBD3-KO cell lines, both HeLa- and HEK293-derived, we observed no ribbon-like or stacked structures; instead, we saw an uncommonly increased amount of vesicles and an enlarged Golgi area, confirming previously published data on an ACBD3-downregulated HeLa cell line by Liao et al. [8]. Taken together, the data demonstrate the indispensability of the ACBD3 protein in Golgi stacking. Unlike in a double knock-out of GRASP-55 and GRASP-65 (Golgi ReAssembly Stacking proteins) [27], the depletion of the Golgi structure in ACBD3-KO cells did not affect the level of GRASP55, GRASP65, and GM130 Golgi proteins, participating in the assembly of the apparatus (Figure 3D,E and Figure A2A,B) nor the glycosylation pattern of the LAMP2 glycoprotein and the level of hexosyl ceramides (Figure 5, Table 1). We showed that defects of Golgi maintenance, induced by depletion of ACBD3 protein, did not affect the glycosylation pattern of LAM2 glycoprotein. Interestingly, another study recently described a disruption of the glycosphingolipid metabolism in an ACBD3-downregulated HeLa cell line [8]. Therefore, it remains unclear whether and to what extent the absence of ACBD3 affects the functions of the Golgi apparatus.

### 3.2. The Role of ACBD3 in Mitochondrial Cholesterol Transport and Mitochondrial Metabolism

To our knowledge, this is the first study focused on the role of ACBD3 in mitochondrial functions. Based on our results from multiple analyses (representation of OXPHOS protein complexes and subunits, mitochondrial respiration, ROS production, mitochondrial ultrastructure, and mtDNA relative quantification) in ACBD3-deficient HEK293 and HeLa cells, the ACBD3 protein is dispensable for proper function of the OXPHOS complexes and its absence has no notable effect on the level of mitochondrial and cellular cholesterol. We assume there is an alternative pathway of transporting cholesterol into mitochondria. In addition to ACBD3, ACBD1 and ACBD2 are also mitochondrial proteins. ACBD1 was discussed previously as a part of the multiprotein complex transporting cholesterol into mitochondria [28,29,30,31], but unlike ACBD3, its role was not described in detail. In C6-2B glioma cells, an ACBD1-dependent formation of mitochondrial pregnenolone was described [32]. The depletion of ACBD1 in MA-10 and R2C Leydig cells led to decreased human chorion gonadotropin-stimulated steroidogenesis and decreased progesterone production, respectively [33,34]. Similarly to ACBD3, ACBD1 also binds TSPO at the outer and inner mitochondrial membrane contact sites and stimulates cholesterol transport into mitochondria. In mitochondria, ACBD1 directly promotes the loading of cholesterol on the CYP11A1 enzyme [35]. Similarly, ACBD2 protein might participate in cholesterol transport into mitochondria. Under ectopic expression of the ACBD2 isoform A, increased basal and hormone-stimulated steroid formation was observed in MA-10 Leydig cells [36]. 

Although most of the research focusing on cholesterol transport into mitochondria has been carried out in the context of steroidogenesis, recently, a new mechanism of mitochondrial cholesterol transport in non-steroidogenic cells has been described [37]. The authors characterized a new protein, Aster-B, which contains a StAR-related transfer domain and a mitochondrial target sequence. Aster-B, together with the Arf1 GTPase, is indispensable for cholesterol transport from ER to mitochondria in C2C12 cells. Depletion of Aster-B or Arf1 leads to a significant decrease in mitochondrial cholesterol content, resulting in mitochondrial dysfunction [37]. 

We were unable to reliably answer the question of if ACBD3 is localized in mitochondria. According to proposed theories, the transduceosome complex probably employs the ACBD3 protein as a tether between TSPO and PKARIα. While the TSPO protein is a transmembrane protein of the OMM, PKA type I is a cytosolic enzyme. Therefore, ACBD3 probably acts as a scaffold between those proteins in close proximity to the OMM, rather than inside the OMM. It is generally accepted that ACBD3 is primarily a Golgi protein, thus its localization in proximity to mitochondria could be transient only after cAMP stimulation. A similar pattern was described in the case of the ACBD2 protein, which is preferentially localized in peroxisomes, but colocalizes with mitochondria upon a cAMP stimulation by dibutyryl-cAMP [36]. Furthermore, we tried to study the impact of ACBD3 protein absence on the other proteins of the transduceosome complex. Unfortunately, our chosen approach did not allow us to uncover the steady-state level of all proteins across both cell types (used antibodies summarized in Table A4).

### 3.3. Lipidomics in ACBD3-KO Cells

We revealed a significantly decreased level of CoQ9 across the ACBD3-KO cell lines by a sensitive mass spectrometry method. In human cells, CoQ9 and CoQ10 are synthetized by the same PDSS1/2 heterotetramer [38] in the first step of the mitochondrial part of the CoQ biosynthesis. To our knowledge, the function of human CoQ9 remains unclear, just as the regulation mechanism specifying if CoQ9 or CoQ10 will be synthesized. Our results suggest that ACBD3 might somehow assist in the regulation of the specificity of PDSS1/2 for chain length formation. A complex study of protein–protein interaction, using BioPlex 2.0, identified the ACAD9 protein as a PDSS1 and PDSS2 interacting partner [39]. The role of ACAD9 in CoQ biosynthesis is not yet known. ACAD9 is also an Acyl-CoA binding protein and seems to have a similar role in the replication of some picornaviruses as ACBD3 [40]. Theoretically, ACAD9 and ACBD3 could have similar but yet unknown functions in the regulation of PDSS1/2. As already mentioned, both CoQ biosynthesis and function in humans still require much research to be carried out. 

ACBD3-deficient cells exhibit a decreased level of SM, together with a normal level of ceramides and hexosyl ceramides, as determined by mass spectrometry. The SMS and GCS activities remain comparable to controls. Taken together, this suggests that ceramide is not effectively transported to the Golgi as a substrate for SMS. Transport of ceramides from ER to the Golgi for the synthesis of SM is CERT (ceramide transport protein)-dependent, but it is known that the CERT pathway does not play a major role in the transport of ceramides for GlcCer synthesis [41,42]. We hypothesize that the decreased level of SM, together with a normal SMS activity in ACBD3-KO cells, could be caused by an impaired transport of ceramides from ER to Golgi. These results are in accordance with previously published data [9], indicating for the first time the role of ACBD3 in the recruitment of PPM1L (ER-resident transmembrane protein phosphatase) to the ER–Golgi membrane contact sites, which seems to be indispensable for the activation of CERT protein. We assume that ACBD3 protein is fundamental in the activation of CERT via PPM1L. How ceramides are delivered from the ER to the site of GlcCer synthesis is unknown [41], but according to our finding, the transport is probably ACBD3 independent. Previously, increased SM and GlcCer levels were observed in an ACBD3-downregulated HeLa cell line [8]. This discrepancy could be related to the amount of ACBD3 residual protein in the downregulated HeLa cell line, as discussed previously [43,44].

## 4. Materials and Methods

### 4.1. Cell Culture 

Human embryonic kidney cells (HEK293, ATCC^®^ CRL-1573™) and HeLa (ATCC^®^ CCL-2™) cells were purchased from the American Type Culture Collection (Rockville, Maryland, MD, USA) and cultivated under standard conditions (37 °C, 5% CO_2_ atmosphere) in high-glucose DMEM (Dulbecco’s Modified Eagle Medium; P04-04510, PanBiotech, Aidenbach, Germany) supplemented with 10% (*v*/*v*) Fetal Bovine Serum (SV30160.03, GE Healthcare, Chicago, IL, USA) and Antibiotic–Antimycotic (XC-A4110/100, Biosera, Nuaille, France).

### 4.2. Preparation of HEK293 and HeLa ACBD3-KO Cell Lines

ACBD3-KO was introduced into HEK293 and HeLa cells by the CRISPR/CAS9 system (Clustered Regularly Interspaced Short Palindromic Repeats). For preparation of ACBD3-KO cells, a commercial plasmid (404320; Santa Cruz Biotechnology, Dallas, TX, USA) was used and in silico analysis of off-target effects revealed a high specificity of all three gRNAs used for the ACBD3 gene. Cells were transfected using Lipofectamine 300 (Invitrogen, Waltham, MA, USA). Then, 24 h after transfection, cells were diluted into a concentration of 5 cells/mL. This suspension was aliquoted (100 µL) into 96-well plates. Wells containing single-cell colonies were identified and further cultivated. Confluent cells in 6-well plates were harvested and characterized by Sodium Dodecyl Sulphate Polyacrylamide Gel Electrophoresis (SDS-PAGE)/Western blot (WB) to confirm complete absence of ACBD3 at the protein level. Cells with no protein levels were sequenced (Sanger sequencing) with the following primers, TGAGTACTTTCAACACTGCATGG, GCCAGACTCACAGTAAAGACAC, GTCAGTTTTCCCTGGGAGCTA, and GTTCTGCAAGTGAACCCCCA, to identify nonsense mutations resulting in premature stop codons. 

### 4.3. Isolation of Mitochondria

For Blue Native Polyacrylamide Gel Electrophoresis (BN-PAGE) analysis, mitochondria were isolated by standard differential centrifugation as described previously [45]. For localization of ACBD3 in the cells and for lipidomic analysis, mitochondria were isolated by Mitochondria Isolation Kit (130-094-532; Miltenyi Biotec, Bergisch Gladbach, Germany).

### 4.4. Electrophoresis and WB

Tricine SDS-PAGE (or glycine SDS-PAGE for LAMP2 detection) was used for separation of proteins according to their molecular weight under denaturing conditions [46]. Cell pellets were resuspended in RIPA buffer (50 mM Tris (pH 7.4), 150 mM NaCl, 1% (*v*/*v*) Triton X-100, 1% (*w*/*v*) sodium deoxycholate, 0.1% (*w*/*v*) SDS, 1 mM EDTA, 1 mM PMSF, and 1% (*v*/*v*) protease inhibitor cocktail), sonicated and lysed for 20 min at 4 °C. The supernatant obtained after lysis and centrifugation was suspended in sample buffer (50 mM Tris (pH 6.8), 12% (*v*/*v*) glycerol, 4% (*w*/*v*) SDS, 0.01% (*w*/*v*) Bromethanol Blue, and 2% (*v*/*v*) mercapthoethanol) to a final concentration of 2–5 µg/µL. A total of 5–15 µg of protein was loaded per lane and separated by 12% (*w*/*v*) polyacrylamide minigels (MiniProtean^®^ 3 System; Bio-Rad, Hercules, CA, USA). BN-PAGE separation [47] was used to analyze the steady-state levels of mitochondrial oxidative phosphorylation system (OXPHOS) protein complexes. The mitochondrial fraction was solubilized with n-dodecyl β-d-maltoside (DDM) at a final 16 mg DDM/mg protein ratio in a buffer containing 1.5 mM aminocaproic acid, 0.05 M Bis-Tris, and 0.5 M EDTA. A total of 15 µg of protein was loaded per lane and separated by 6–15% (*w*/*v*) polyacrylamide gradient gels (MiniProtean^®^ 3 System; Bio-Rad).

SDS-PAGE and BN-PAGE gels were transferred onto Immobilon-P PVDF Membrane (Millipore, Burlington, MA, USA) by semi-dry electroblotting using the Hoefer semi-dry transfer unit (Hoefer, Harvard Bioscience, Holliston, MA, USA) or Trans-Blot Turbo Transfer System (Bio-Rad).

For immunodetection, membranes were incubated for 2 h in primary antibodies at room temperature (RT) or overnight at 4 °C in 2% non-fat milk. Particular antibodies for individual experiments are summarized in Table A3. All membranes were detected with peroxidase-conjugated secondary antibodies and SuperSignal™ West Femto Maximum Sensitivity Substrate or SuperSignal™ West Pico PLUS Chemiluminescent Substrate (34,096 and 34,577, respectively; Thermo Fisher Scientific, Waltham, MA, USA) using G:Box (Syngene, Cambridge, UK) and analyzed by Quantity One software (Bio-Rad).

### 4.5. High-Resolution Respirometry

HEK293 and HeLa cells were cultivated to approximately 80% confluence, harvested by incubation (5 min, 37 °C) with TE (Trypsin, 0.05% (*w*/*v*); EDTA (0.02%, *w*/*v*)), washed and suspended in mitochondrial respiration medium MiRO5 kit (60101-01, Oroboros Instruments, Innsbruck, Austria), and centrifuged (5 min, 300 g, 24 °C). Cells were resuspended in approximately 500–800 µL MiRO5 and then counted by a Handheld Automated Cell Counter (Millipore). Two million cells were added in a 2 mL chamber with preheated (37 °C) MiR05 medium and measured in the Oroboros O2k-FluoRespirometer. After cells addition, ROUTINE respiration was analyzed, which is a physiological respiration controlled by cellular energy demand, energy turnover, and the degree of coupling to phosphorylation. Next, ATP synthase inhibitor, oligomycin (25 nM), was added to inhibit the mitochondrial respiration and investigate proton leak. This non-phosphorylating state is a respiration maintained mainly to compensate for the proton leak at a high chemiosmotic potential. Afterwards, the FCCP uncoupler (1 µM titration steps, final conc. 7–10 µM) was added to obtain the maximal electron transfer capacity, meaning oxygen consumption in the noncoupled state at optimum uncoupler concentration. In the electron transfer state, the mitochondrial membrane potential is almost fully collapsed and provides a reference state for flux control ratios. Finally, inhibitors of complex I and III, antimycin A (2.5 µM) and rotenone (0.5 µM), respectively, were added to obtain residual oxygen consumption (ROX), which is due to oxidative side reactions remaining after the inhibition of the electron transfer pathway in cells. ROX state was used as a respiratory and methodological correction factor for other respiratory states. Flux control ratio (*FCR*) is the ratio of oxygen flux in respiratory control states, normalized for maximum flux in a common reference state, to obtain theoretical lower and upper limits of 0.0 and 1.0. *FCR* provides an internal normalization and express respiratory control independent of mitochondrial amount and shows the quality of mitochondrial respiration [48].

### 4.6. Analysis of mtDNA Content

The relative amount of mtDNA was analyzed by real-time PCR (RT-PCR) as described previously [49]. Briefly, total DNA was isolated from cells using the QIAamp DNA Mini Kit (51306, QIAGEN, Hilden, Germany) according to the manufacturer’s instructions. To quantify the mtDNA content, 16S rRNA was used as a mitochondrial target and the GAPDH gene as a nuclear target. Primer sequences were published previously, and PCR conditions were as follows: initial denaturation at 95 °C for 15 min, 42 cycles of 95 °C for 15 s, annealing at 54 °C for 20 s and elongation at 72 °C 30 s, and a final elongation at 72 °C for 7 min using StepOnePlusTM (Applied Biosystems, Foster City, CA, USA). Ten-fold serial dilutions of the genomic DNA (from 100 to 10 ng) from control cell lines were included in each run to generate the calibration curve. The nuclear target was used to quantify nuclear DNA in order to normalize the amount of mtDNA per cell.

### 4.7. Flow Cytometry Measurement of DHE Stained Cells

Measurements of dihydroethidium (DHE)-stained cells by flow cytometry were performed as described previously [50]. In brief, 5 × 10^5^ cells per sample were stained by 10 μM DHE (D23107, Invitrogen) for 30 min at 37 °C and measured by BD FACS CANTO II flow cytometer (BD Biosciences, San Jose, CA, USA) with the FACSDiva Version 6.1.3. software. As a positive control (increased ROS production), 100 μM menadione (M5625, Sigma, St. Louis, MI, USA) was used.

### 4.8. Confocal and Transmission Electron Microscopy (TEM)

For confocal microscopy, where indicated, cells were stained by 200 nM MitoTracker^®^ Red CMXRos (M7512, Invitrogen) for 30 min at 37 °C before fixation. The cells were fixed in 4% PFA, permeabilized by 0.1% Triton-X100, blocked in 10% iFBS (1 h, RT), and labeled overnight by specific antibodies (summarized in Table A3). Incubations with specific secondary antibodies were performed the next day. Mounted cells (P36931, Invitrogen) were captured by confocal microscope Leica SP8X, image acquisition using HC PL APO 63x/1.40 OIL CS2 objective, and HyD detectors with gating set to 0.3–6 ns (Leica Microsystems, Wetzlar, Germany).

Measurements of relative Golgi area were performed using ImageJ 1.48 v (Wayne Rasband, National Institutes of Health, Bethesda, Maryland, USA) and correlation coefficients of GM130 and TGN46 signals were determined by the LAS X software (Leica Microsystems). To assess statistical significance, a Mann–Whitney test was calculated by GraphPad Prism version 8.3.0 for Windows (GraphPad Software, San Diego, CA, USA).

For TEM analysis, the cells were fixed using a modification of Luft’s method [51]. The cells were incubated in PBS containing 2% potassium permanganate for 15 min, washed with PBS, and dehydrated with an ethanol series. The cells were subsequently embedded in Durcupan Epon (Electron Microscopy Sciences, Hatfield, PA, USA), sectioned on an Ultracut microtome (Reichert, Depew, NY, USA) to thicknesses ranging from 600 to 900 Å, and finally stained with lead citrate and uranyl acetate. A Jeol JEM 1400 Plus transmission electron microscope (JEOL, Tokyo, Japan) was used for image acquisition.

Mitochondria of a normal size and cristae formation were counted as ‘normal’, and mitochondria with atypical ultrastructure were counted as ‘abnormal’. Overall, more than 300 mitochondria from 27 TEM pictures were categorized. Disputable mitochondria were excluded from the analysis. Significance was determined by Mann–Whitney t-test, using GraphPad Prism.

### 4.9. Lipidomics

For the lipidomics analysis, two types of input material were used—cells and mitochondria. The cellular material was obtained from a confluent 6-well plate (in triplicate for each ACBD3-KO clone), rinsed twice with PBS, scraped into 1 mL PBS, and stored at 80 °C for a downstream analysis. In the case of mitochondria, the organelles were isolated by the Mitochondrial Isolation Kit (Miltenyi Biotec) from 10^7^ cells. One sample for each ACBD3-KO clone was used. Overall, ACBD3-KO mitochondria were analyzed in quadruplicate. For a quantification of absolute values from the MS analysis, protein concentration of each sample was used (BCA assay). 

Samples were processed via LC−MS workflow LIMeX (LIpids, Metabolites, and eXposome compounds) for simultaneous extraction of complex lipids, polar metabolites, and exposome compounds that combines an LC−MS untargeted and targeted analysis. The extraction of metabolites was carried out using a biphasic solvent system of cold methanol, methyl tert-butyl ether (MTBE), and 10% methanol [52,53,54,55]. 

Due to repeated measurements, linear mixed-effects models with interactions were used to analyze the data. The subject of the patient was considered as a random effect. *p*-values less than 5% were considered as statistically significant. Analyses were conducted using the R statistical package version 3.6.3. (R Core Team (2020), Vienna, Austria).

### 4.10. Sphingomyelin Synthase Activity

The activity of sphingomyelin synthase (SMS) was measured as described previously [23]. In short, 1.5 × 10^6^ cells were incubated with 2.5 μM C6-NBD-ceramide (144090, Abcam, Cambridge, UK) for 1 h at 37 °C. Lipids were extracted by the Folch method and separate by thin-layer chromatography (TLC). The same amount of protein (determined by BCA assay) was used for individual spotted samples. Visualization of the fluorescence-labeled sphingolipid species was performed by G:box (Syngene) and quantified by Quantity One software (Bio-Rad). 

## 5. Conclusions

Herein, using ACBD3-KO HEK293 and HeLa cells, we confirm the importance of the ACBD3 protein in Golgi structure maintenance, but the consequences of its absence on proper Golgi function remain unclear. We observed a decreased level of SM, together with a normal SMS activity and ceramide level, which we suggest to be caused by an impaired transport of ceramides from ER to Golgi (CERT pathway). Further analysis is required to confirm our hypothesis. We also show that in HEK293 and HeLa cells, ACBD3 is not vital for cholesterol homeostasis in mitochondria or for the function of the mitochondrial oxidative phosphorylation system. We speculate about the existence of an alternative mechanism for mitochondrial cholesterol transport, independent of ACBD3. One such mechanism could be the recently described novel pathway of cholesterol transport [37]. Altogether, the exact mechanism of cholesterol transport into mitochondria remains largely unknown, and further investigation is required to gain proper insight.

## Figures and Tables

**Table 1:** Statistical analysis of lipidomics data from 5A–5H.

		Chol	CE	CoQ9	SM	CER	HexCer	Hex2Cer	Hex3Cer
WT vs. KO	cells	NS	**	**	***	NS	NS	NS	NS
	mitochondria	NS	***	***	**	NS	NS	NS	NS
	HeLa	NS	***	***	NS	NS	NS	NS	NS
	HEK293	NS	NS	***	***	NS	NS	NS	NS
	overall	NS	***	***	***	NS	NS	NS	NS

*p* < 0.01 (**); *p* < 0.001 (***); NS: not significant. Abbreviations: CE: cholesteryl esters, CER: ceramides, Chol: cholesterol, CoQ9: coenzyme Q_9_, HexCer: hexosylceramides, Hex2Cer: dihexosylceramides, Hex3Cer: trihexosylceramides, KO: knock-out, SM: sphingomyelins, WT: wild type.

## Data Availability

The data presented in this study are available upon request from the corresponding author.

## Appendix A

**Table A1:** Expression level of ACBD3 protein in different organs and tissues according to The Human Protein Atlas (available on 28 May 2021 at https://www.proteinatlas.org/).

Level of Expression	Organs/Tissues	
high	digestive system	duodenum, small intestine, colon, gallbladder, pancreas, and appendix
	brain	cerebral cortex and hippocampus
	others	prostate, placenta, and bone marrow
medium	male tissues	testis, epididymis, and seminal vesicles
	female tissues	vagina, fallopian tube, endometrium, cervix, uterine, and breast
	endocrine tissues	thyroid and adrenal gland
	brain	cerebellum and caudate
	digestive system	salivary gland, esophagus, stomach, and rectum
	lung	nasopharynx, bronchus, and lung
	others	kidney and urinary bladder, smooth muscle, skin, lymph node, and tonsil
low	others	adipose and soft tissues, liver, ovary, skeletal muscle, spleen, and oral mucosa

**Table A2:** Expression level of ACBDs proteins according to MitoCarta 2.0.

	Training Dataset	MSMS NUM TISSUES **
ACBD1	Possible mito *	12/14
ACBD2	Mito	14/14
ACBD3	Possible mito *	0
ACBD4	Non mito	0
ACBD5	Non mito	0
ACBD6	Non mito	0
ACBD7	Non mito	0

* Indicating evidence based on NCBI GO mitochondrial annotation or MitoP2 database, but not in mito. ** Number 0–14 tissues where gene products were found by MS/MS.

**Table A3:** Summary of antibodies used in experiments.

Figure	Antibody	Company	Catalog Number	Dilution	Method
1A	ACBD3	Atlas Antibodies	HPA015594	1:2000	tricine SDS-PAGE
	β-actin	Cell Signaling Technology	4970	1:2000	tricine SDS-PAGE
	OPA1	BD Transduction Laboratories	612607	1:1000	tricine SDS-PAGE
	SERCA2	Abcam	2861	1:1000	tricine SDS-PAGE
	GM130	Sigma	G7295	1:3000	tricine SDS-PAGE
1B	ACBD3	Atlas Antibodies	HPA015594	1:500	ICC
	Giantin	Abcam	37266	1:200	ICC
	SERCA2	Abcam	2861	1:200	ICC
3A	GM130	Abcam	52649	1:250	ICC
	TGN46	Biorad	AHP500G	1:250	ICC
3D, A2A	ACBD3	Atlas Antibodies	HPA015594	1:2000	tricine SDS-PAGE
	GM130	Abcam	52649	1:5000	tricine SDS-PAGE
	GRASP65	Abcam	174834	1:1000	tricine SDS-PAGE
	GRASP55	ProteinTech	10598-1-AP	1:5000	tricine SDS-PAGE
	GAPDH	Abcam	8245	1:13,000	tricine SDS-PAGE
	β-actin	Cell Signaling Technology	4970	1:2000	tricine SDS-PAGE
3E, A2B	LAMP2	Santa Cruz Biotechnology	18822	1:500	glycine SDS-PAGE
4A, 4B	NDUFA9	Abcam	14713	1:1000	BN-PAGE
	SDH70	Abcam	14715	1:10,000	BN-PAGE
	CORE2	Abcam	14745	1:8000	BN-PAGE
	COX2	Abcam	110258	1:10,000	BN-PAGE
	ATPβ	Abcam	14730	1:4000	BN-PAGE
4C, 4D	ACBD3	Atlas Antibodies	HPA015594	1:2000	tricine SDS-PAGE
	NDUFA9	Abcam	14713	1:2500	tricine SDS-PAGE
	NDUFB6	Abcam	110244	1:4000	tricine SDS-PAGE
	SDH70	Abcam	14715	1:20,000	tricine SDS-PAGE
	SDH30	Abcam	14714	1:1000	tricine SDS-PAGE
	CORE1	Abcam	110252	1:5000	tricine SDS-PAGE
	CORE2	Abcam	14745	1:20,000	tricine SDS-PAGE
	COX1	Abcam	14705	1:4000	tricine SDS-PAGE
	COX2	Abcam	110258	1:10,000	tricine SDS-PAGE
	COX5a	Abcam	110262	1:2000	tricine SDS-PAGE
	ATPβ	Abcam	14730	1:2000	tricine SDS-PAGE
	VDAC1	Abcam	14734	1:2000	tricine SDS-PAGE
	GAPDH	Abcam	8245	1:13,000	tricine SDS-PAGE
	α-tubulin	Cell Signaling Technology	2125	1:2000	tricine SDS-PAGE
A1	ACBD3	Atlas Antibodies	HPA015594	1:500	ICC

Abbreviations: ICC—immunocytochemistry, SDS-PAGE—sodium dodecyl sulphate polyacrylamide gel electrophoresis, BN-PAGE—blue native polyacrylamide gel electrophoresis.

**Table A4:** Summary of antibodies used in experiments.

	Antibody	Company	Catalog Number	Results from SDS-PAGE in HEK293/HeLa
Not shown	StAR	Abcam	58013	↓↓↓/↓↓↓
	VDAC1	Abcam	14734	not changed/not changed
	TSPO	Cell Signaling	9530	↓↓↓/not changed
	ACBD1	Atlas Antibodies	HPA051428	↓↓↓/↓↓↓
		Abcam	16806	↓↓↓/not changed
	ATAD3	Abcam	112572	↓↓↓/NA

Annotation: ↓↓↓—low specificity of the antibody; NA—not analyzed.
